# On-chip detection of non-classical light by scalable integration of single-photon detectors

**DOI:** 10.1038/ncomms6873

**Published:** 2015-01-09

**Authors:** Faraz Najafi, Jacob Mower, Nicholas C. Harris, Francesco Bellei, Andrew Dane, Catherine Lee, Xiaolong Hu, Prashanta Kharel, Francesco Marsili, Solomon Assefa, Karl K. Berggren, Dirk Englund

**Affiliations:** 1 Department of Electrical Engineering and Computer Science, Massachusetts Institute of Technology, 77 Massachusetts Avenue, Cambridge, Massachusetts 02139, USA; 2 Research Laboratory of Electronics, Massachusetts Institute of Technology, 50 Vassar Street, Cambridge, Massachusetts 02139, USA; 3 Department of Electrical Engineering, Columbia University, 1300 South West Mudd, MC4712, 500 West 120th Street, New York, New York 10027, USA; 4 Jet Propulsion Laboratory, California Institute of Technology, 4800 Oak Grove Drive, Pasadena, California 91109, USA; 5 IBM TJ Watson Research Center, Yorktown Heights, New York 10598, USA

## Abstract

Photonic-integrated circuits have emerged as a scalable platform for complex quantum systems. A central goal is to integrate single-photon detectors to reduce optical losses, latency and wiring complexity associated with off-chip detectors. Superconducting nanowire single-photon detectors (SNSPDs) are particularly attractive because of high detection efficiency, sub-50-ps jitter and nanosecond-scale reset time. However, while single detectors have been incorporated into individual waveguides, the system detection efficiency of multiple SNSPDs in one photonic circuit—required for scalable quantum photonic circuits—has been limited to <0.2%. Here we introduce a micrometer-scale flip-chip process that enables scalable integration of SNSPDs on a range of photonic circuits. Ten low-jitter detectors are integrated on one circuit with 100% device yield. With an average system detection efficiency beyond 10%, and estimated on-chip detection efficiency of 14–52% for four detectors operated simultaneously, we demonstrate, to the best of our knowledge, the first on-chip photon correlation measurements of non-classical light.

## Abstract (web summary)

The integration of single-photon detectors, as superconducting nanowire single-photon detectors, in photonic-integrated circuits is a goal of quantum information science. Here, Najafi *et al.* introduce a micrometer-scale flip-chip process enabling such a integration in a scalable way.

## Graphical abstract

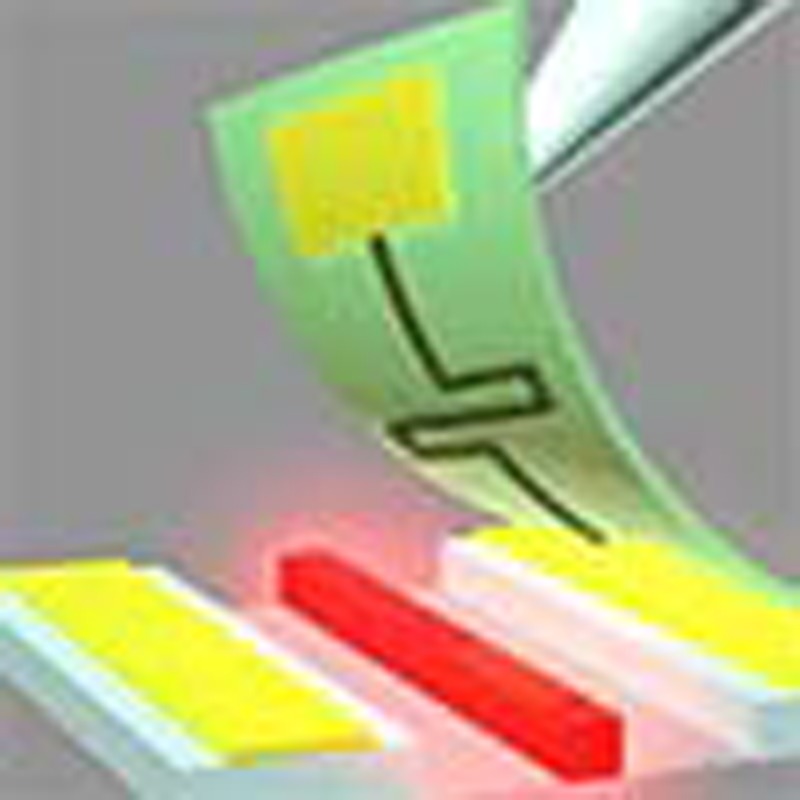

Photonic-integrated circuits (PICs) are being developed for a wide range of applications in quantum information science, including quantum simulation^1^,^2^,^3^,^4^, quantum photonic state generation^5^,^6^,^7^,^8^, quantum-limited detection^9^ and linear optical quantum computing^10^,^11^,^12^,^13^. These applications require multiple detectors with low timing jitter (TJ). The lowest TJ for infrared photon detection has been achieved with superconducting nanowire single-photon detectors (SNSPDs^14^,^15^) based on sub-100-nm-wide and 3- to 6-nm-thick niobium nitride (NbN) nanowires^16^. However, to date there has been no scalable approach for integrating SNSPDs into PICs: while single, isolated waveguide-integrated SNSPDs have been demonstrated^17^,^18^,^19^,^20^, the highest reported system detection efficiency (SDE) for just two SNSPDs integrated into the same photonic circuit remains significantly below 1%^21^,^22^. The central challenge when building systems with multiple SNSPDs remains the low fabrication yield, which is limited by defects at the nanoscale^23^. This yield problem is exacerbated when such detectors are integrated onto photonic chips, which can require tens of additional fabrication steps of their own.

Here we report on a micrometer-scale flip-chip process developed to overcome the yield problem by separating the PIC and the SNSPD fabrication processes. Using this method we show scalable integration of low-jitter SNSPDs with silicon and aluminum nitride (AlN) waveguides. With four on-chip detectors operated simultaneously, we demonstrate the first on-chip correlation measurements of non-classical light. Our approach is compatible with a wide range of PICs and other substrates, including complementary metal oxide semiconductor (CMOS)-compatible silicon photonic platforms, in a back-end-of-the-line step. This demonstration, along with recent progress on scalable on-chip sources^5^, enables fully integrated photonic processors for quantum information science.

## Results

### Integration with silicon PICs

**Figure 1:**
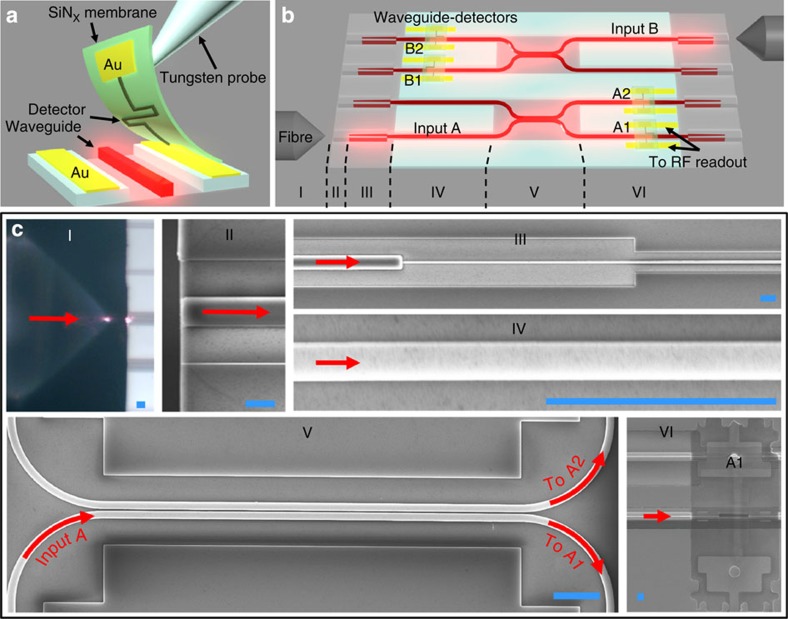
Assembly of high-system-efficiency PIC with integrated detectors via membrane transfer. (**a**) Membrane transfer of an SNSPD onto a photonic waveguide. (**b**) Sketch of photonic chip with four waveguide-integrated detectors (A1, A2, B1 and B2). (**c**) Micrographs of sections I–VI labelled in **b**. Infrared light (red arrows) was coupled from a lensed fibre (I) with a spot diameter of 2.5 μm into a 2 × 3 μm polymer coupler (II). The coupler overlapped with a 50- to 500-nm-wide inverse-tapered section of a silicon waveguide (III). The input light travelled along the 500-nm-wide waveguide (IV) over a distance of 2 mm before reaching a 50:50 beam splitter (directional coupler in V) followed by the waveguide-integrated detectors (VI). The equivalent length of the scale bar (blue) is 3 μm.

Figure 1a outlines the elements of the assembly process. Hairpin-shaped SNSPDs^17^,^18^,^24^ were fabricated on ~200-nm-thick silicon nitride (SiN_*x*_) membranes; silicon-on-oxide (SOI) PICs were fabricated separately (see Methods). After evaluating the SNSPDs in a cryostat, high-performance detectors were selected from the fabrication chip and transferred onto high-performance SOI waveguides. Using this method, we assembled a proof-of-concept photonic circuit, shown in Fig. 1b, comprising an optical network with two input ports and four output ports, each coupled to an SNSPD. We measured an estimated on-chip detection efficiency (ODE) up to 52±6% for 1,550-nm-wavelength single photons and TJ as low as 42 ps. The light was coupled into the waveguides using inverse tapered couplers with 3.7±0.3 dB insertion loss (see Methods and ref. 25), resulting in a SDE (from the external fiber) up to 19±2%. This system efficiency enables the first on-chip intensity autocorrelation measurements of non-classical light, demonstrated here for photon pairs generated by spontaneous parametric downconversion (SPDC).

**Figure 2:**
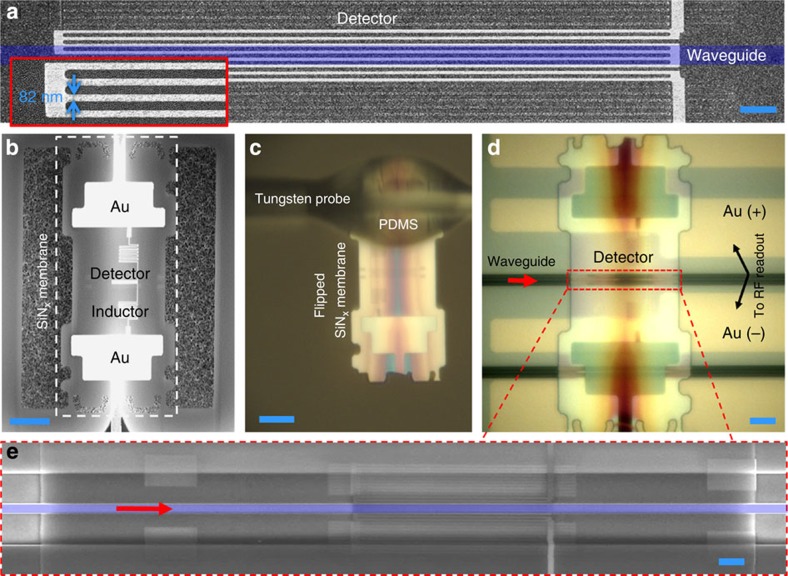
Detector assembly process. (**a**) Scanning Electron Micrograph (SEM) of an SNSPD based on 82-nm-wide superconducting nanowires (see inset). The purple strip marks the intended location of the waveguide after the integration is complete. The equivalent length of the scale bar (blue), 1 μm. (**b**) SEM of suspended SiN_*x*_ membrane with detector on top. The area of the membrane was 50 × 120 μm. The equivalent length of the scale bar (blue), 20 μm. (**c**) The detector was removed from the carrier chip using a tungsten microprobe containing a drop of hardened PDMS near the tip. The membrane was then flipped and the detector aligned to the waveguide under an optical microscope; this step simultaneously established electrical contact to Au strips on the photonic chip. The equivalent length of the scale bar (blue), 20 μm. (**d**) Optical micrograph of an SNSPD integrated with a Si waveguide. The equivalent length of the scale bar (blue), 10 μm. (**e**) SEM of waveguide-integrated detector in the region marked by a dashed line in **d**. The silicon waveguide is highlighted in purple. The equivalent length of the scale bar (blue), 1 μm.

The detector comprised multiple nanowires connected in parallel (see Supplementary Fig. 1), as shown in Fig. 2a. This SNSPD variant^26^,^27^ has been shown to double the signal-to-noise ratio of the photodetection voltage compared with traditional single-wire SNSPDs. The detector length was designed using a finite-element model^24^ to ensure optical absorption exceeding 50% (see Supplementary Fig. 2 and Supplementary Methods). We present simulations in Supplementary Fig. 3 showing that (1) detector-to-waveguide misalignment on the scale of the nanowire pitch and (2) scattering at the SiN_*x*_ membrane interface both affect efficiency by <1%.

We fabricated 225 detectors on a 200- to 300-nm-thick SiN_*x*_ layer over a Si substrate. The underlying silicon was then etched (see Methods), leaving hundreds of free-standing membranes carrying SNSPDs. One of these suspended membranes is shown in Fig. 2b. Each membrane was connected to the bulk substrate through six narrow bridges, two of which connected the detector on the membrane electrically to large contact pads on the bulk substrate for testing the detectors after the etch step (see Supplementary Fig. 4c).

We characterized all detectors to identify low-jitter, high-efficiency devices (typically about 30% of the detectors). As shown in Fig. 2c, we removed selected detector membranes from the substrate using tungsten microprobes coated with polydimethylsiloxane (PDMS) adhesive. We then placed membranes detector-side-down onto the target waveguide with sub-1 μm alignment accuracy under an optical microscope. For electrical readout, the gold pads on the membranes contacted complementary pads on the PIC (Fig. 2d). These gold–gold contacts withstood repeated thermal cycles with no noticeable degradation (see Supplementary Discussion). Figure 2e shows the resulting waveguide-integrated detector. Because we transferred only high-performance detectors, we were able to achieve perfect yield in the assembled device, resolving the non-scalability of low-jitter SNSPD fabrication^23^.

### On-chip detection of photon pairs

The detection of photon pairs on a chip requires the controllable integration of multiple high-efficiency single-photon detectors within the same circuit. Using the process outlined in the previous section, we integrated four detectors (labelled A1, A2, B1 and B2) on a PIC and characterized the performance of the assembled system shown in Fig. 1b,c using four parameters: SDE, ODE, full width at half maximum TJ and noise-equivalent incident power (NEIP). The SDE includes all losses (that is, coupling and transmission) between the fibre port outside the cryostat and the detector. We determined the SDE from the ratio of the SNSPD photocount rate (PCR) to the photon flux coupled into the fibre port (see Methods). This yields an SDE of 19±2% for input A (11% for A1 and 8% for A2) and 7±1% for input B (3% for B1 and 4% B2). These SDE values represent an improvement by 2 orders of magnitude compared with previous approaches for multi-detector integration^21^.

**Figure 3:**
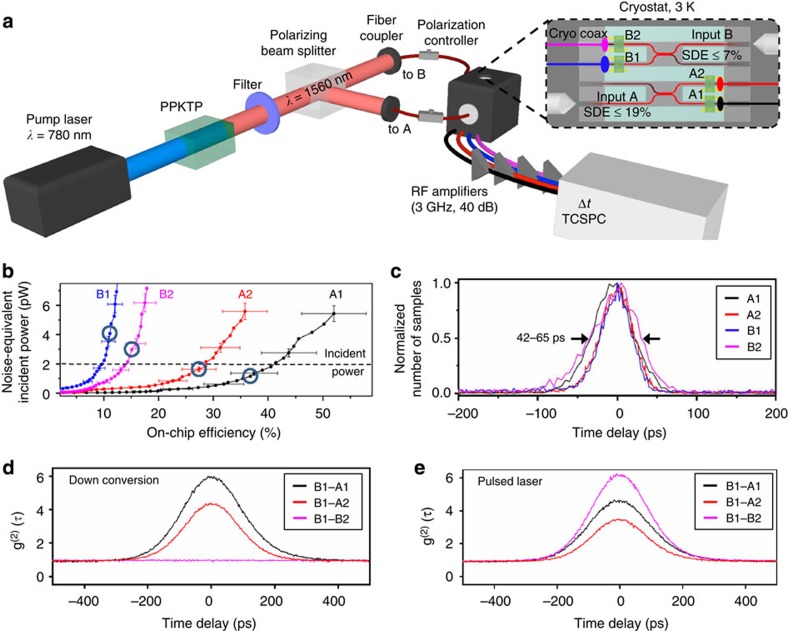
On-chip detection of photon pairs. (**a**) Experimental set up for on-chip 
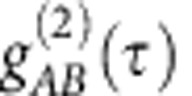
 measurements of an entangled-photon source coupled into the PIC (cooled to 3 K). (**b**) Noise-equivalent incident power versus on-chip efficiency for the detectors shown in Fig. 1b. The circles mark the operation points chosen for subsequent coincidence measurements. The error bars, shown for selected data points, denote a pessimistic estimate of the standard error (see Methods). (**c**) Photodetection delay histogram of the detectors shown in Fig. 1b when operated at the maximum on-chip efficiency. (**d**,**e**) Coincidence counts versus time delay between B1 and {A1, A2, B2} for the entangled-photon-pair source (**d**) and for a mode-locked sub-picosecond-pulsed laser (**e**). The average laser power was adjusted to match that of the photon-pair source. The data was acquired with a time-correlating counter (TCSPC, HydraHarp 400).

The ODE is defined as the probability that a photon already coupled into the waveguide is detected^18^,^21^ (see Methods). We estimated the ODE as SDE/*η*_c_, where *η*_c_=0.22 accounts for coupling losses into the PIC (3.69 dB) and the splitting ratio of the directional couplers before the SNSDPs (3 dB). The transferred detectors reached ODEs between 14±2% and 52±6% and 42- to 65-ps TJ (see Fig. 3b,c). The *η*_c_ values were obtained from a series of PIC transmission measurements at room temperature outlined in the Methods section. We note that, since the fiber coupling in the cryostat was performed with slip-stick stepper stages with worse resolution than room-temperature piezo scanners used to estimate *η*_c_=0.22 and its error, the low-temperature *η*_c_ is expected to be smaller than room-temperature value, and the ODE values provided here are pessimistic.

The NEIP is given by SDCR/SDE·*ħω*, where SDCR is the system dark count rate and *ħω*=0.81 eV. Figure 3b shows the NEIP versus ODE for the waveguide detectors on couplers A and B. The ratio of the power incident onto the detectors (IP) and the NEIP characterizes the signal-to-noise ratio for single-shot measurements. In this work, the NEIP was limited by radiation leakage (see Supplementary Discussion and ref. 27) through a cryostat window used to image and align the lensed fibres to the polymer couplers (Fig. 1c–I). Hence, for subsequent measurements, we operated the detectors at lower ODEs of 12±1% to 37±4% (circled points in Fig. 3b), which reduced the dark count rate (~800 k counts per second, on the same order as the PCR) and resulted in a IP/NEIP ratio of ~0.5–1.7. The low NEIP of these detectors is crucial for characterizing picowatt-level optical signals, which can be the case for non-classical light sources.

We used these high-SDE SNSPDs to characterize time-energy entangled-photon pairs entirely on the PIC. Photon pairs were generated by type-II SPDC from a 1 cm periodically poled potassium titanyl phosphate (PPKTP) waveguide, as shown in Fig. 3a. Signal and idler photons of ~1 ps duration and orthogonal polarization were separated using a polarizing beam splitter and sent into inputs A and B of the PIC. The SPDC pump power was adjusted to generate pairs at ~1.5 × 10^8^ Hz, corresponding to a multi-pair probability of ~4 × 10^−4^ over the correlation timing uncertainty of 200 ps. We obtained the second-order correlation function from 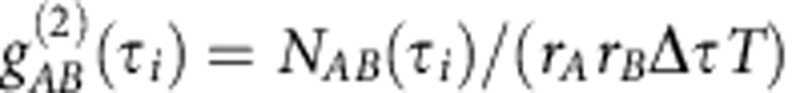, where *N*_AB_ (*τ*_*i*_) is the measured number of coincidences between inputs A and B at time difference *τ*_*i*_, *r*_A_ (*r*_B_) is the count rate from input A (B), Δ*τ* is the coincidence bin duration and *T* is the integration time. Figure 3d shows the resulting 
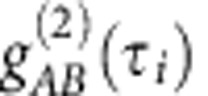
 function. Photon bunching is evident between inputs A and B, but not within individual channels (that is, between A1 and A2 or B1 and B2), as expected for a photon pair source. The observed peak heights of 
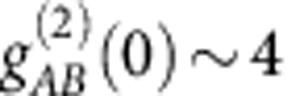
 and 
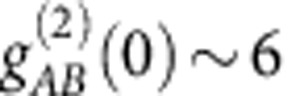
 are lower than the theoretical value of infinity for ideal detectors due to the finite IP/NEIP ratio of our detectors (see Methods) and the non-zero multi-pair probability. By contrast, when pulses from a mode-locked laser were injected into inputs A and B with an average photon number per pulse >1, bunching was observed between all detector pairs (Fig. 3e), as expected for a pulsed classical light source.

### Large-scale integration of on-chip detectors

**Figure 4:**
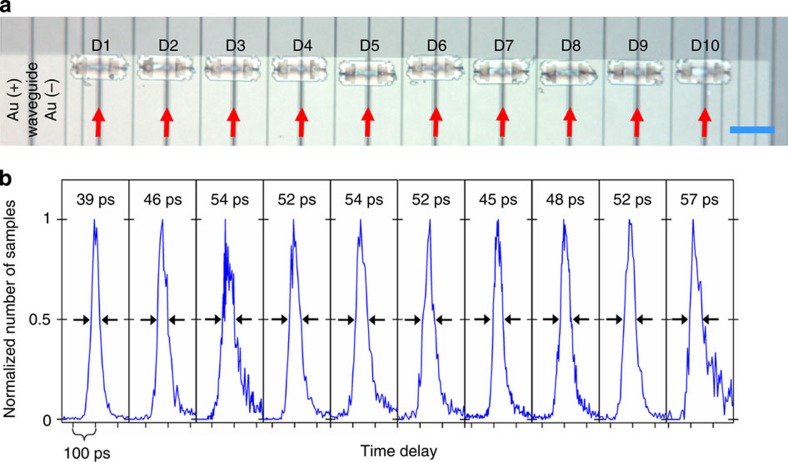
Array of adjacent waveguide-integrated low-jitter detectors. (**a**) Optical micrograph of 10 waveguide-integrated detectors D1–10 assembled on the same photonic chip. The waveguides are marked by red arrows. The equivalent length of the scale bar (blue), 100 μm. (**b**) Top-illuminated photodetection delay histogram of D1–10 measured in a cryogenic probe station at 2.8 K base temperature. The timing jitter is listed above each histogram.

The ability to pre-select functioning single-photon detectors enables scaling to more detectors with unity yield. We define yield as the ratio of detectors that operate in the high-efficiency single-photon regime (also referred to as avalanche regime, see ref. 27). In this regime the detectors show sub-100-ps TJ (ref. 27). Figure 4a shows 10 SNSPDs (D1–10) on adjacent waveguides with TJ values of 39–57 ps for 1,550-nm-wavelength light. For rapid characterization, these devices were measured by top illumination in a cryogenic probe station. The photodetection delay histograms for all detectors are shown in Fig. 4b.

### Integration with different material systems

**Figure 5:**
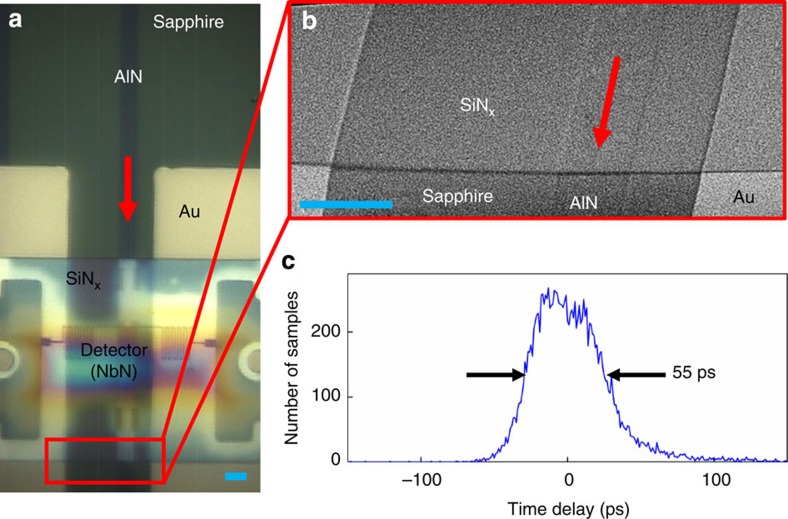
Integration of nanowire single-photon detectors with new material systems. (**a**) Single-photon detector integrated with a multi-mode AlN-on-sapphire waveguide. The equivalent length of the scale bar (blue), 5 μm. (**b**) Angled SEM showing the membrane conforming to waveguide and Au pad surfaces. The equivalent length of the scale bar (blue), 5 μm. (**c**) Top-illuminated photodetection delay histogram of the detector shown in **a**,**b**.

The SNSPD integration method presented here can be applied to many different substrates. As an example, Fig. 5a shows an SNSPD integrated with an AlN-on-sapphire waveguide, also showing good jitter performance (Fig. 5c). The AlN-on-sapphire material system has several distinguishing physical properties from SOI, including a wide transparency window and high piezoelectric transduction efficiency^28^. The ease of SNSPD integration on AlN waveguides suggests that the same method would also apply to other materials, such as lithium niobate, where traditional efforts at detector integration^29^ have proven challenging. Furthermore, the membrane transfer process could be used to integrate other electro-optic devices, such as III–V lasers or single-photon sources, onto PICs, therefore enabling the ground-up assembly of a quantum (photonic) circuit using pre-selected high-performance components.

Since the device membrane is flexible, it conforms to the target chip, even if that chip is not perfectly flat (see Fig. 5b). Because of the small size of the membrane, the process is also relatively tolerant to defects on the target chip, as opposed to processes involving large-area flip-chip bonding (for example, see ref. 30), which require both surfaces to be free of defects.

## Discussion

The system efficiency of these devices could be improved with several changes to the PIC. Propagation loss could be reduced from 2–3 dB cm^−1^ to 0.3 dB cm^−1^ using ridge waveguides^31^. On-chip coupling, loss can also be reduced from 3.7 dB using either high-performance grating couplers, which can achieve 0.6 dB loss^32^, or edge couplers, which can achieve 1 dB (ref. 33). In the cryostat, fibre-to-chip coupling losses could be improved using piezo scanners or by permanently bonding the chip to the fibre. Scattering at the SiN_*x*_ membrane edge is small (<1%), but can be improved by making this transition in a wider region of the Si waveguide, where the evanescent field above the waveguide would be reduced. Last, the absorption into the SNSPD increases with device length; a tapered waveguide with stronger evanescent overlap can also lead to greater absorption. An optical cavity could be used to increase the detector-waveguide interaction length, but at the loss of bandwidth. As shown experimentally in the Supplementary Discussion, an increase in detector coupling length from 17 to 28 μm increases system efficiency by 26±3% for a PIC geometry similar to Fig. 1.

The system dark count rate could be reduced significantly by eliminating the cryostat windows, though without optical access this would entail fibre bonding to the PIC. In fact, eliminating the windows reduced the operation-point dark count rate of our chip from ~800 k counts per second to <5 k counts per second (see Supplementary Fig. 14b). The TJ of the on-chip detectors can be further improved to 33 ps by decreasing the loss in the RF transmission lines, as shown in the Supplementary Discussion. On-chip amplification electronics, for example ref. 34, could be used to further reduce jitter to 24 ps. To speed up the manual assembly process currently employed, a high-throughput assembly process could be adopted^35^.

In conclusion, we have demonstrated the scalable integration of high-performance SNSPDs into PICs. We assembled 10 adjacent waveguide-integrated detectors on a silicon PIC with 100% yield and observed detector TJ values between 39 and 57 ps. Waveguide-integrated SNSPDs on the same PIC enabled on-chip *g*^(2)^(*τ*) measurements of non-classical light. Scaling to many tens to hundreds of detectors would ultimately be limited by the readout complexity. There is ongoing work to address this problem using electrical multiplexing schemes^36^. For more detectors, which require greater bandwidth, optical wavelength division multiplexing could be used, employing high-speed (>50 GHz) modulators already available on PICs^37^. The integration process demonstrated here is CMOS compatible; indeed, the silicon PICs used in this experiment were fabricated in a CMOS compatible process with the exception of the polymer waveguide couplers, which can be replaced with SiN_*x*_ (ref. 38). Thus, it appears likely that tens to hundreds of SNSPDs and other heterogeneous circuit elements can be integrated into high-performance PICs. This demonstration opens the door to fully integrated, high-performance photonic processors for quantum information science.

## Methods

### Detector fabrication

A SiN_*x*_ layer (typically 200- to 300-nm-thick) was grown via plasma-enhanced chemical vapour deposition on double-polished silicon substrates. The NbN film was deposited on top of the SiN_*x*_ layer via reactive magnetron sputtering (AJA system) at a substrate holder temperature of 800 °C. The sheet resistance of the 4-nm-thick NbN films (thickness estimated from the deposition time) was 515 Ω per square and the critical temperature was 10.9 K. Electrical contact pads were defined by ultraviolet exposing a 700-nm-thick polydimethylglutarimide layer covered with 1.5-μm-thick photoresist (S1813) for 13 s at 2,300 μW cm^−2^ and developing the bilayer for 24 s in CD-26. This process achieved an undercut of the photoresist by 1 to 2 μm, enabling smooth gold pad edges after liftoff. Ti (10 nm) and Au (15 nm) were evaporated and the liftoff was performed in acetone under sonication for 2 min followed by a 1-min dip in CD-26 and a 1-min de-ionized water (DI) dip. Seventy-nanometer-thick electron-beam-resist (hydrogen silsesquioxane) was spun on top of the sample, exposed in a 30 keV electron beam lithography tool (Raith 150, exposure dose 700–850 μC cm^−2^) and developed in tetramethylammonium hydroxide (TMAH) at 27 °C for 3 min. The hydrogen silsesquioxane pattern was transferred into NbN via a 2.5-min CF_4_ reactive-ion etch at 50 W. To improve electron-beam dose uniformity^39^, additional features were exposed outside the hairpin-shaped detector. These dummy structures, also referred to as proximity-effect-correction features, are shown as parallel lines in dark grey outside the detector in Fig. 2a.

### Detector suspension

The suspension process is outlined in Supplementary Fig. 5. The detector was covered with S1813 and a trench pattern was exposed in the photoresist. This pattern was then used as an etch mask to define trenches around the detector through the SiN_*x*_ layer via reactive-ion etch with CF_4_. This trench pattern left the underlying silicon substrate exposed. The silicon under the SiN_*x*_ layer was removed using XeF_2_, a selective isotropic etch gas. In the final step, the photoresist was removed in an N-methyl-2-pyrrolidone solution (see Supplementary Methods), resulting in a detector on a suspended SiN_*x*_ membrane.

### Transfer probe preparation

PDMS was mixed in a 10:1 ratio with the curing agent and allowed to set for 4 h. A tungsten microprobe (Ted Pella Autoprobe 100) was dipped in the PDMS solution, resulting in a PDMS droplet near the tip of the probe. The PDMS-covered probe was baked on a hot plate at 100 °C for 8 h, followed by sonication in an ethanol–water mixture (see ref. 40).

### Membrane pickup and alignment accuracy

To remove the detector membranes from the substrate, three of the six microbridges connecting the membranes to the substrate (shown in Fig. 2b) were broken using a plain tungsten probe. A probe was then placed under the membrane and used to bend the membrane upwards, as shown in Supplementary Fig. 6b. A second tungsten probe, covered with PDMS droplet and mounted on a six-axis micromanipulator, was then used to lift the membrane from the substrate, touching only the passive (back) side of the membrane (Supplementary Fig. 6c). The PDMS served as an adhesive surface during the transfer (Supplementary Fig. 6d) from the fabrication (carrier) chip to the PIC chip, where the membrane was then rotated, aligned and placed down under an optical microscope (Supplementary Fig. 6e). After placement, the PDMS probe was used to press down on any regions of the membrane that exhibited interference fringes, indicating a separation between the PIC and membrane. Crucially, the detector surface was not in contact with any PDMS or other surfaces during membrane pickup, minimizing contamination risk. Supplementary Figure 7 shows detectors aligned to silicon waveguides on a photonic chip using the alignment marks highlighted in red. The arrows in Supplementary Fig. 7a mark the boundaries between which the waveguide must be located for efficient detection. Of the four membrane detectors placed, all were aligned to the waveguide. Efficient detection requires close contact between the detector and the waveguide. Interference fringes serve as an indicator of the closeness-of-contact. The detector shown in Supplementary Fig. 8a has little to no interference fringes visible, implying close contact between the membrane and waveguide chip surface. The opposite is true for the micrograph shown in Supplementary Fig. 8b. Here we can see visible fringing in the central region above the waveguide as well as near the gold pads. The detector shown in Supplementary Fig. 8b would, in the best case, have poor detection efficiency and electrical properties. In the worst case it would exhibit no response to stimuli.

### PIC fabrication

The PIC was fabricated on a 10 Ω cm, p-doped, 200-mm SOI wafer from SOITEC. The wafer had a 220-nm-thick silicon device layer on top of a 2 μm buried oxide layer. The 500-nm-wide silicon waveguides were fabricated on a CMOS line at the IBM Watson Research Center using electron-beam lithography. In a subsequent optical lithography step, SU8 polymer couplers were fabricated to allow 3.7 dB coupling loss from a lensed fibre to the silicon waveguide (see ref. 41 for further details). The gold pads on the PIC were fabricated in a similar manner to that outlined in the detector fabrication section above.

### TJ measurements

We used a mode-locked, sub-picosecond-pulse-width laser emitting at 1,550 nm wavelength and 38 MHz repetition rate. The laser output was split into two SMF28 fibres, which we coupled to the detector under test and to a low TJ InGaAs photodiode (Thorlabs S1R5). The light coupled to the detector was attenuated to <5 pW and operation of the detector in single-photon regime was checked by confirming the linearity of the photocount rate as a function of incident photon flux (see Supplementary Fig. 9b). For detectors A1, A2, B1 and B2 the light was coupled to the waveguides A and B using a lensed fibre as shown in Fig. 1b–I. The second sample, containing detectors D1–10, was back-illuminated with a high-NA fibre with light from the mode-locked laser, and single-photon operation regime was confirmed as described above. The electrical output from the detector and from the photodiode were sent to a 6-GHz-bandwidth, 40 G samples per second oscilloscope. We measured time delay *t*_D_ between the detector pulse (start signal) and the pulse from the fast photodiode (stop signal). We acquired the instrument response function (IRF), a histogram of >2,000 samples of *t*_D_, and measured the TJ of the detector, which was defined as the full width at half maximum of the IRF.

### Detection efficiency measurements and error estimates

A schematic depiction of the experimental set up used to measure the SDE of the waveguide-integrated SNSPDs is shown in Supplementary Fig. 10. Light from a fibre-coupled CW laser (Thorlabs S3FC1550, emitting at *λ*=1,550 nm, output power 1 mW) was split into two outputs using a calibrated, fibre-coupled 50/50 splitter (Thorlabs 10202A-50-FC). One output, used to monitor the power directly, was coupled to an InGaAs photodiode (Thorlabs S154C), calibrated with a NIST-traceable curve down to 100 pW input power. Light in the second output passed through a variable attenuator (JDS Uniphase HA9, manually calibrated), a polarization controller and an SMF28 fibre feedthrough to couple to the PIC in the cryostat. The calibration of the HA9 beyond the sensitivity of the photodiode was confirmed as follows: we recorded the SNSPD count rate under a given HA9 attenuation value (typically 50–80 dB), then replaced the HA9 with fixed fibre optic attenuators of the same attenuation value. The fixed fibre optic attenuators used here—Thorlabs FA attenuators connected in series, with an attenuation value of 10 to 25 dB per unit—were calibrated at high laser power using the InGaAs photodiode. The detector count rate measured with the HA9 set to a given nominal attenuation value was within *δ*=10% (relative error) of the count rate measured at the same attenuation value set with the FA attenuators. Since the SDE includes all losses in the system, except for the variable attenuator, the overall relative error of the SDE value can also be estimated as *δ*_SDE_≈*δ*. The measured SDE for detectors A1, A2, B1 and B2 and corresponding error bars are shown in Supplementary Fig. 9a. The error on the on-ODE *δ*_ODE_ is naturally larger than *δ*_SDE_ due to uncharacterizable defects in the on-chip structures. We extracted the on-ODE as ODE=1/*η*_fibre*–*WG_·1/*η*_DC_ (SDE per detector), where *η*_fibre*–*WG_ is the fibre-to-waveguide coupling efficiency and *η*_DC_ is the on-chip transmission of 47±4.6% due to the nominal 3 dB splitting ratio of the on-chip directional coupler (beam splitter). *η*_fibre*–*W_ and *η*_DC_ were estimated from room-temperature measurements using high-precision scanning piezos (as opposed to the stepping cryogenic piezos used in our cryostat), and the results are shown in Supplementary Fig. 11. From these measurements, the on-chip coupling loss and propagation loss were calculated from a linear regression, resulting in an estimated loss due to coupling on and off the chip of 7.372±0.39 dB. Assuming defects in the structures for on- and off-chip coupling are uncorrelated, we estimate the distribution of the coupling efficiency for each coupler to be 43±3%. All other on-chip losses are included in the ODE estimate (for example, we do not normalize the ODE to account for ~2.15 dB cm^−1^ propagation loss in the waveguide nor do we normalize to account for the loss in the polarization controller shown in Supplementary Fig. 10) and therefore they do not contribute to the ODE error. The overall ODE error is estimated as 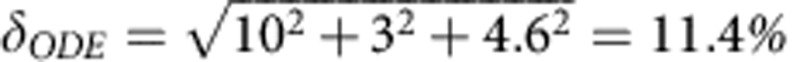. We confirmed that the detector operated in single-photon regime during the system efficiency measurements, as demonstrated by the linearity of the photodetection count rate versus incident photon flux shown in Supplementary Fig. 9b.

### Optical absorption and critical current

Before transferring detectors, we measured the room-temperature transmission of the silicon waveguides using lensed fibres identical to those used in the cryostat. However, the fibres were mounted on high-precision piezo scanning stages, which are more precise than the stepper stages used in the cryostat. After transferring the detectors onto these waveguides, we measured the room-temperature transmission again to obtain the amount of light either scattered or absorbed by the detector. Our simulations indicate that the absorption is far stronger than the scattering. The measured values were 74%, 74%, 65% and 62% for A1, A2, B1 and B2, respectively, with errors typically <5%. We note that the error and expected value of these transmission measurements were not used, and therefore do not contribute to the ODE calculations; we performed these measurements simply to confirm intimate detector-to-waveguide contact before proceeding to later rounds of testing. The photonic chip was then mounted into a closed-cycle cryostat and the detectors operated at 3 K base temperature. The critical currents after detector undercut and transfer (15.2 μA, 16.8 μA, 16.4 μA and 14.8 μA) were about 20% lower compared with pre-undercut values measured on the solid silicon substrate, possibly arising from the small thermal capacitance of the membranes (as noted in the Supplementary Discussion).

### Photon pair generation

We used a PPKTP waveguide source to generate photons pairs at 1,561 nm wavelength via type-II SPDC. A 50 mW pump beam at 781 nm was focused on a PPKTP waveguide with cross-section 2 × 4 μm. The waveguide was defined by ion implantation, and was 1 cm long. The phase matching bandwidth was ~1.5 nm, and the generated photon pair flux was estimated to be 1.5 × 10^8^ pairs per second. The down-converted signal and idler photons were coupled into a single fibre and split with a fibre polarizing beam splitter. The output fibres were coupled to polarization controllers, which were connected in turn to the fibres leading into the cryostat.

### Correlation measurements


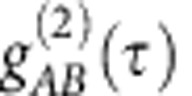
 can be calculated from experimental data using the formula given in the main text. To incorporate detector dark counts, we define rates 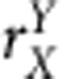, where *X*ε{A, B} (for channels A and B, respectively) and *Y*ε{P, D} (corresponding to a ‘photon’ and ‘dark count,’ respectively). 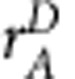, for example, is the rate at which channel A registers dark counts, and 
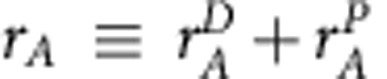
 is the count rate on channel A. Now 
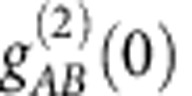
 is


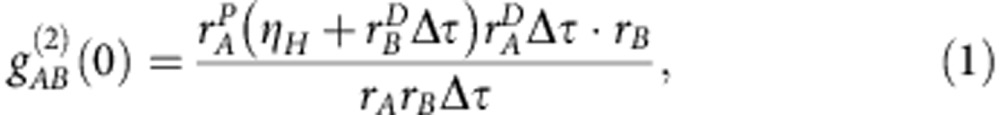


where *η*_H_ is the probability that channel B registers a photon given that channel A also registers a photon (that is the heralding efficiency) and Δ*τ* is the bin duration. For 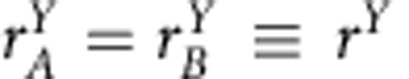 and the ratio 
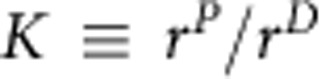
,


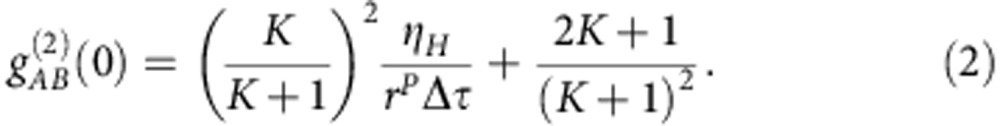


In our experiment, 
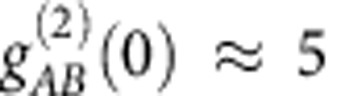
, which gives an estimate of the heralding efficiency, *η*_H_=4 × 10^−3^.

## Author contributions

F.N., J.M., S.A., K.K.B. and D.E. conceived and designed the experiments. F.N., F.B., J.M., X.H., F.M., D.E. and K.K.B. designed the detectors. F.N. and A.D. fabricated the detectors. J.M., S.A. and D.E. developed the waveguide chip. C.L. and J.M. developed the SPDC source. F.N. and J.M. performed the experiments. N.C.H., J.M., X.H., P.K. and D.E. developed the membrane transfer process. N.C.H. performed the membrane transfer. F.N., J.M., N.C.H., K.K.B. and D.E. prepared the manuscript.

## Additional information

**How to cite this article:** Najafi, F. *et al.* On-chip detection of non-classical light by scalable integration of single-photon detectors. *Nat. Commun.* 6:5873 doi: 10.1038/ncomms6873 (2015).

## Supplementary Material

Supplementary Information Supplementary Figures 1-15, Supplementary Table 1, Supplementary Discussion, Supplementary Methods and Supplementary References
