# Supplementary material for: On-chip detection of non-classical light by scalable integration of single-photon detectors
Source: Nat Commun. 2015 Jan 9;6:5873. doi: 10.1038/ncomms6873 (PMC4354051; doi:10.1038/ncomms6873)
Supplement: Supplementary Information — Supplementary Figures 1-15, Supplementary Table 1, Supplementary Discussion, Supplementary Methods and Supplementary References [file ncomms6873-s1.pdf]

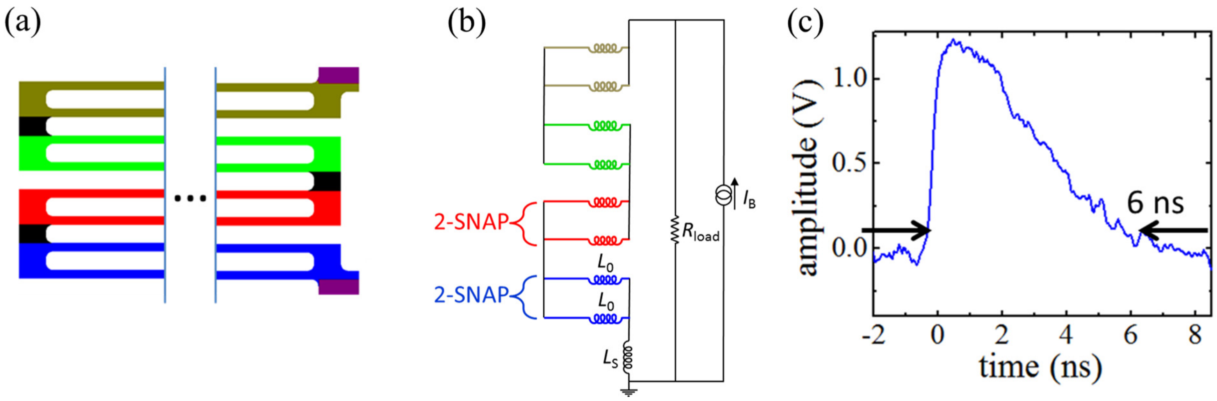


**Supplementary Figure 1.** (a) Sketch representing the nanowire arrangement of the detector shown in Figure 2(a) in the main text. The detector consisted of four units in series, each comprising two parallel nanowires. (b) Equivalent circuit diagram for the detector. (c) Measured single-shot voltage trace of the output pulse of a detector with the geometry shown in (a, b).

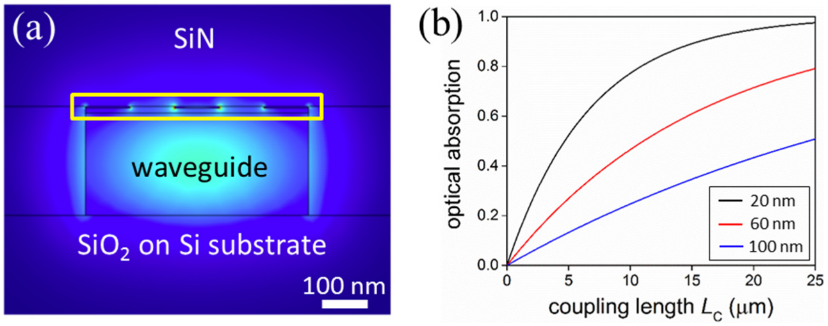


**Supplementary Figure 2.** (a) Cross-sectional geometry of waveguide-integrated detector (marked in yellow) superposed by the simulated spatial distribution of the intensity of the waveguide eigenmode. The detector consists of 80-nm-wide, 4-nm-thick NbN nanowires arranged in a 200 nm pitch. The 500-nm-wide silicon waveguide was designed for 1550 nm center wavelength. (b) Calculated optical absorption in the detector vs. coupling length for a residual resist thickness of 20, 60 and 100 nm.

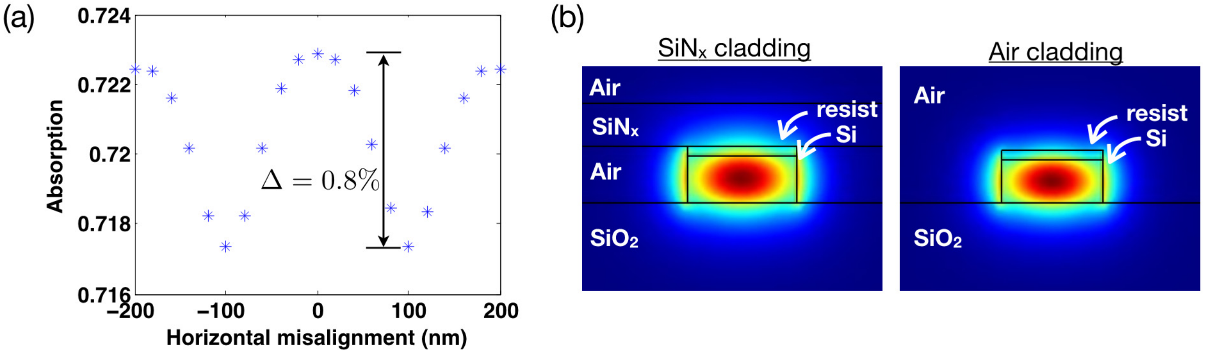


**Supplementary Figure 3.** Imperfections in the detector-membrane integration. (a) Simulation of the absorption of the detector given in-plane misalignment orthogonal to the direction of light propagation. This simulates the tolerance of the detector performance to misalignment smaller than the size of the detector. The absorption is decreased by at most 0.8%. (b) Magnitude of the electric field of the fundamental TE mode of the waveguide with (left) and without (right) cladding of the waveguide by the $SiN_x$ membrane. Black lines were drawn on the figure to more clearly display the simulated device geometry.

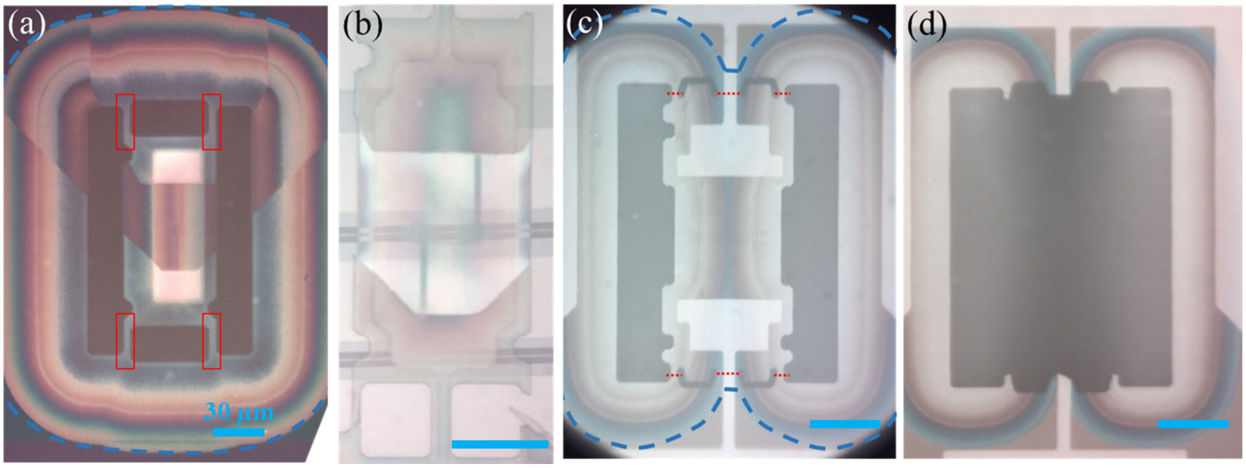


**Supplementary Figure 4.** Top-down optical micrographs of membrane-detectors. The scale bar in blue represents 30 µm in equivalent length. (a) Suspended membrane held with long microbridges (enclosed with red lines) and surrounded by four large trenches. The dashed blue lines separate the undercut $SiN_x$ region from the bulk substrate. (b) Transferred membrane with a design similar to the membrane shown in (a). (c) Suspended membrane with only two large trenches and short microbridges with constrictions. (d) Remaining structures on the primary $SiN_x$ chip after the membrane identical to the membrane shown in (c) has been removed.

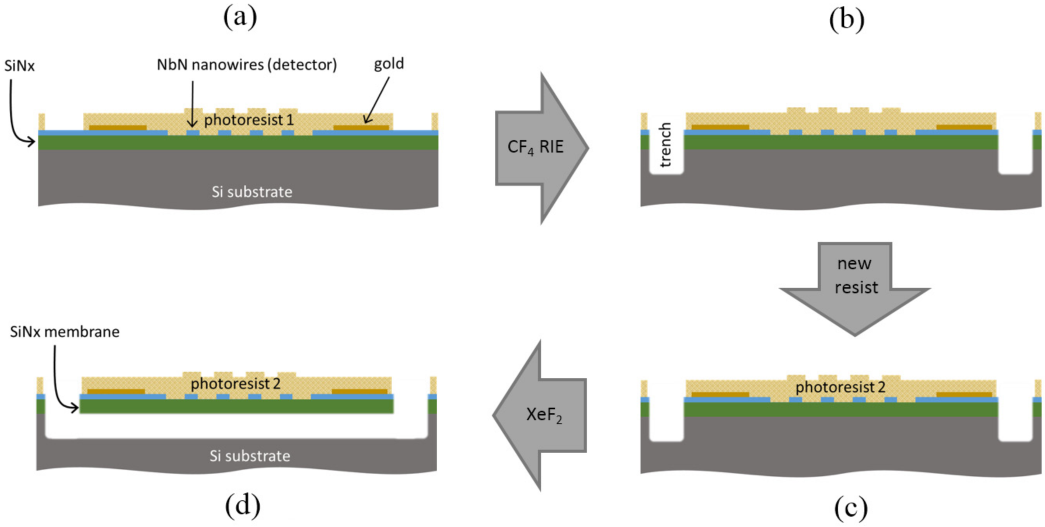


**Supplementary Figure 5.** Schematic cross-section illustrating the membrane-detector fabrication process.

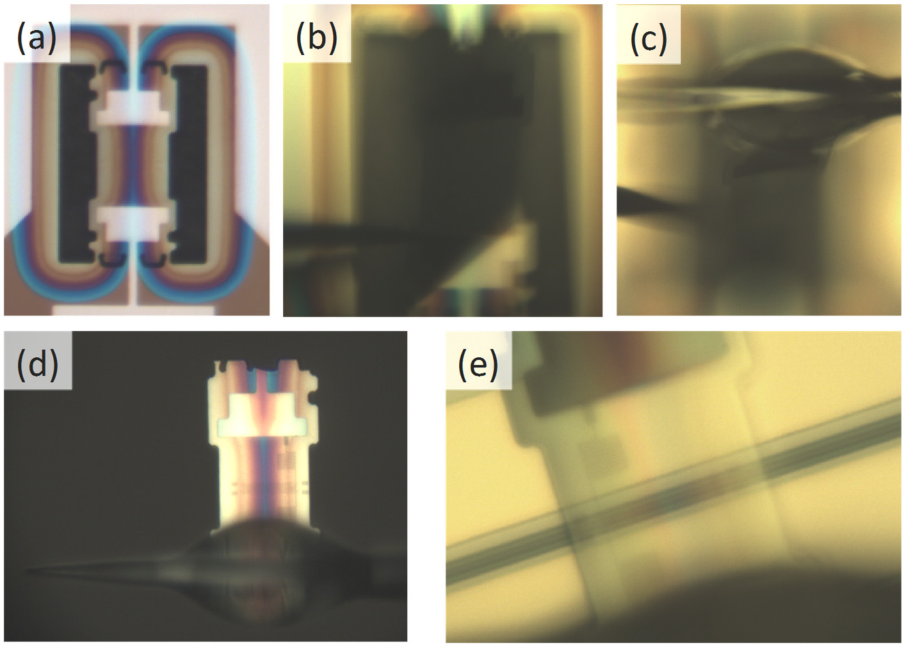


**Supplementary Figure 6.** Optical micrographs of SNSPD transfer process steps.

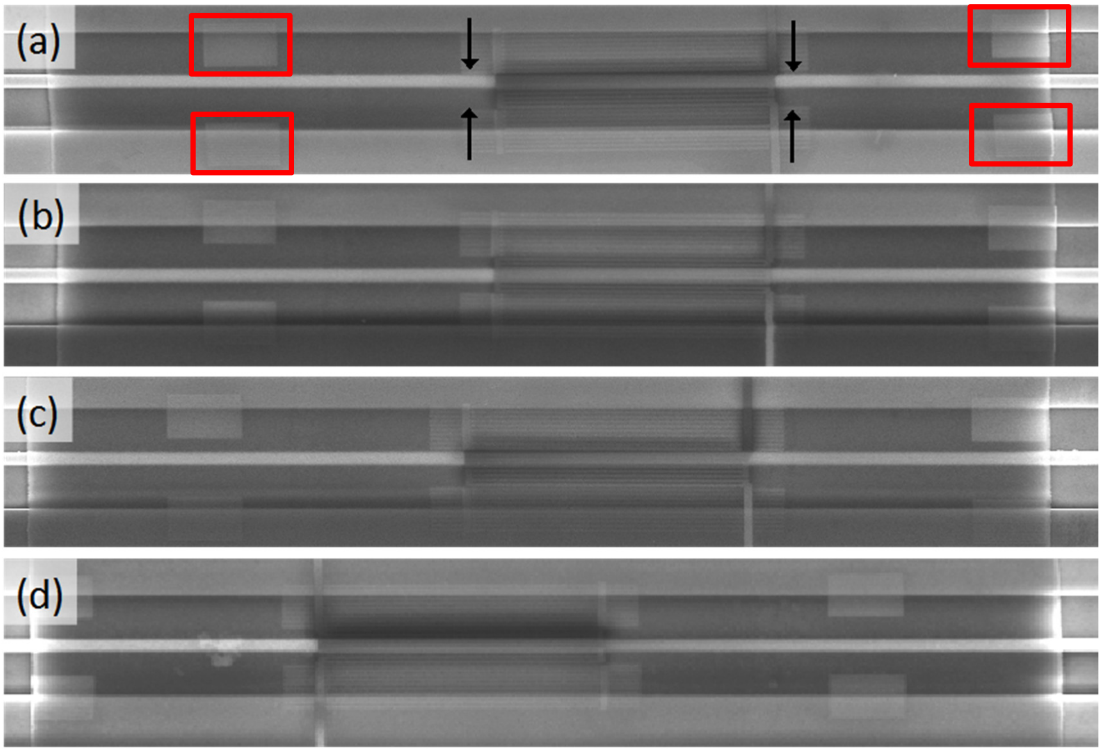


**Supplementary Figure 7.** SEM of four detectors (out of a total of four transfers) aligned to 500-nm-wide waveguides.

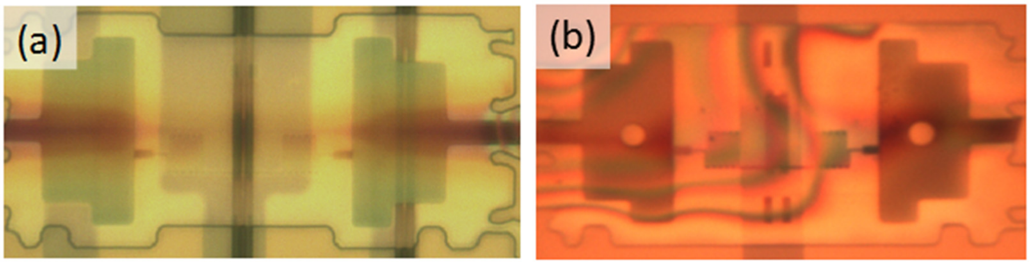


**Supplementary Figure 8.** Optical micrographs of two different membrane-detectors after transfer. (a) Membrane-detector with negligible interference fringes. (b) Membrane-detector with visible fringing indicating poor contact.

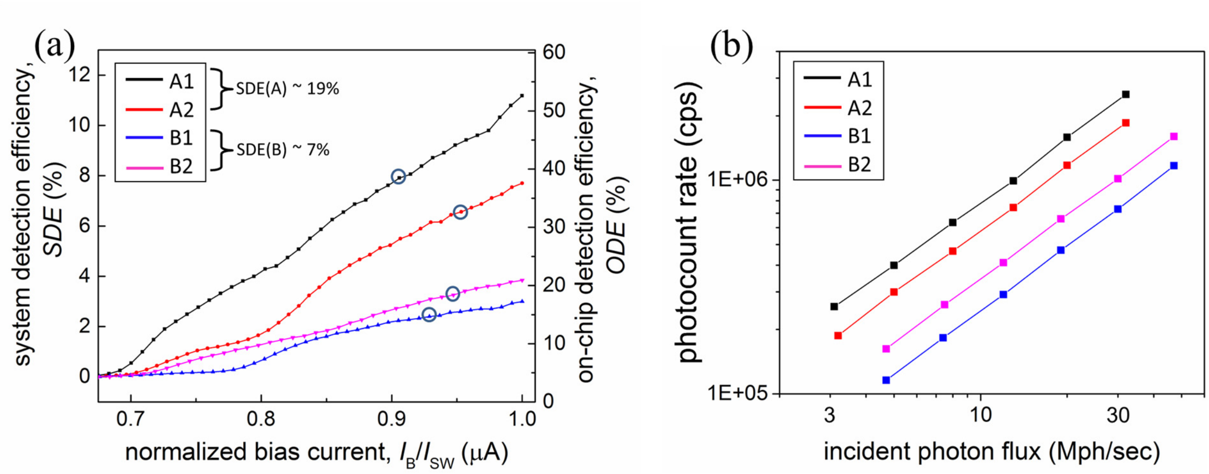


**Supplementary Figure 9.** (a) System detection efficiency (SDE) vs. normalized bias current of the waveguide-integrated detectors shown in Figure 1 in the main text. The bias current ($I_B$) on the horizontal axis was normalized by the maximum bias current (switching current $I_{SW}$) of the detector. The relative error of the SDE value is ±10% and the relative error of the ODE values is ±11.4%. (b) Photocount rate in counts per second vs. incident photon flux for the detectors A1, A2, B1 and B2. The detectors were biased at the operation point marked by circles in (a). For the measurements shown in Figures 3(b-e) in the main text the average photon flux was kept at ~10-15 million photons per second, which was well within the single-photon regime of the detectors.

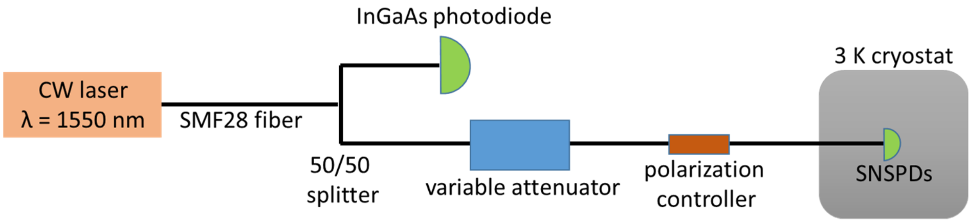


**Supplementary Figure 10.** Schematic depiction of experimental setup used to measure the system detection efficiency of waveguide-integrated detectors.

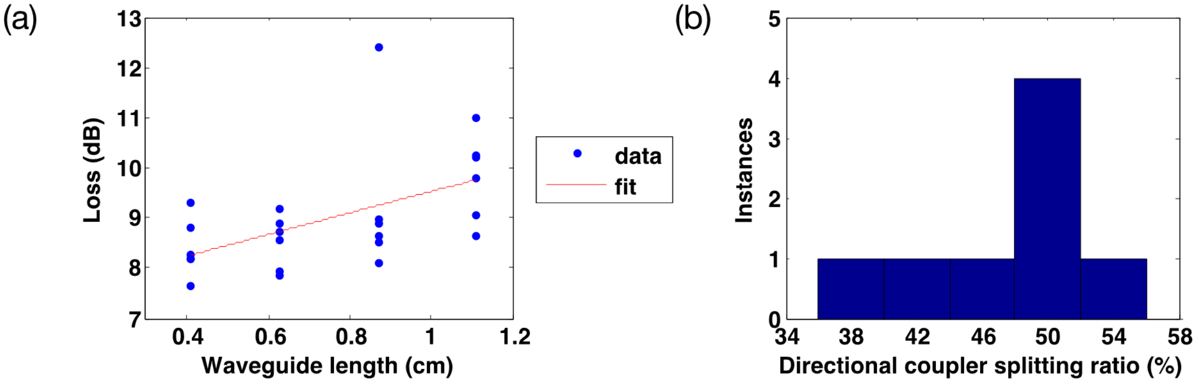


**Supplementary Figure 11.** Distributions of on-chip waveguide components. (a) Propagation loss of single-mode silicon waveguides of different lengths. Extrapolation to the zero length value gives two times the on-chip coupling loss. The slope of the line gives the propagation loss in the waveguide. (b) Histogram of the on-chip splitting ratios of the directional couplers measured at room temperature.

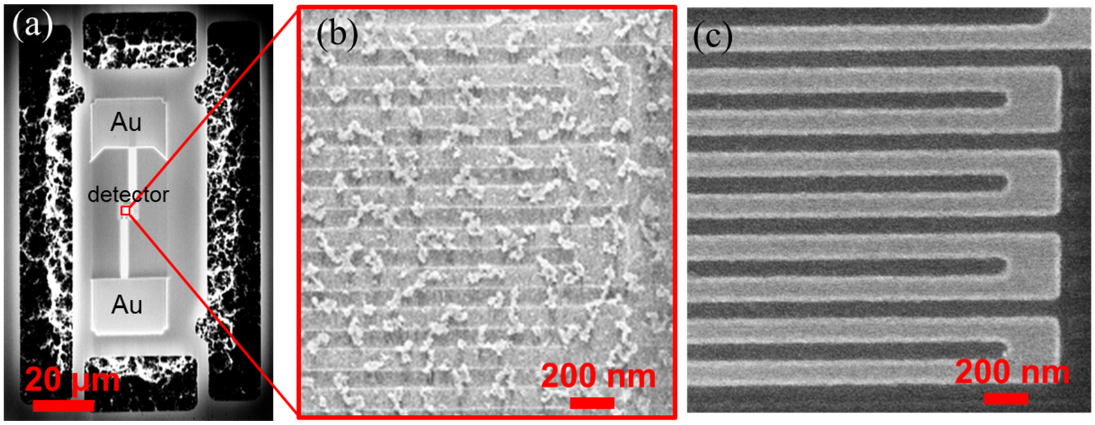


**Supplementary Figure 12.** (a, b) SEMs of membrane-detector after the protective photoresist was stripped in an oxygen plasma. (c) SEM of membrane-detector after the photoresist was stripped in an NMP solution.

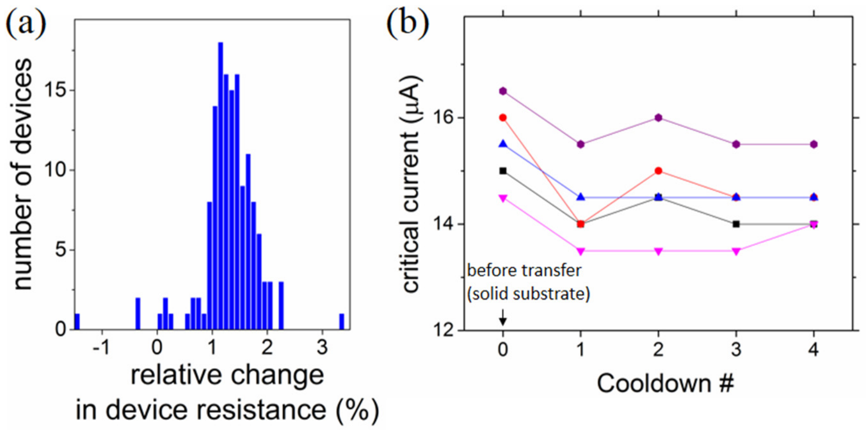


**Supplementary Figure 13.** (a) Histogram of relative change in room-temperature detector resistance after membrane undercut compared to the resistance values before membrane undercut (suspension). (b) Critical current of detectors that were successfully transferred onto a secondary substrate on ~300-nm-thick $SiN_x$ membranes. Up to four thermal cycles were performed between ~2.8 K and room temperature.

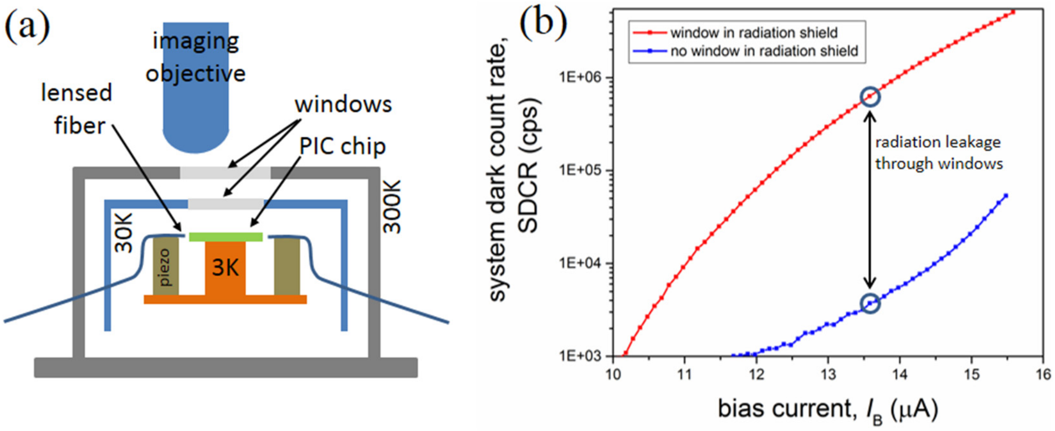


**Supplementary Figure 14.** (a) Schematic cross-section of cryostat used to operate the PIC chip. (b) System dark count rate (SDCR) curves representative of waveguide-integrated detectors operated in the cryostat shown in (a). The red curve shows the SDCR during the regular operation of the cryostat with windows, and the blue curve shows the SDCR with the windows replaced with copper plates.

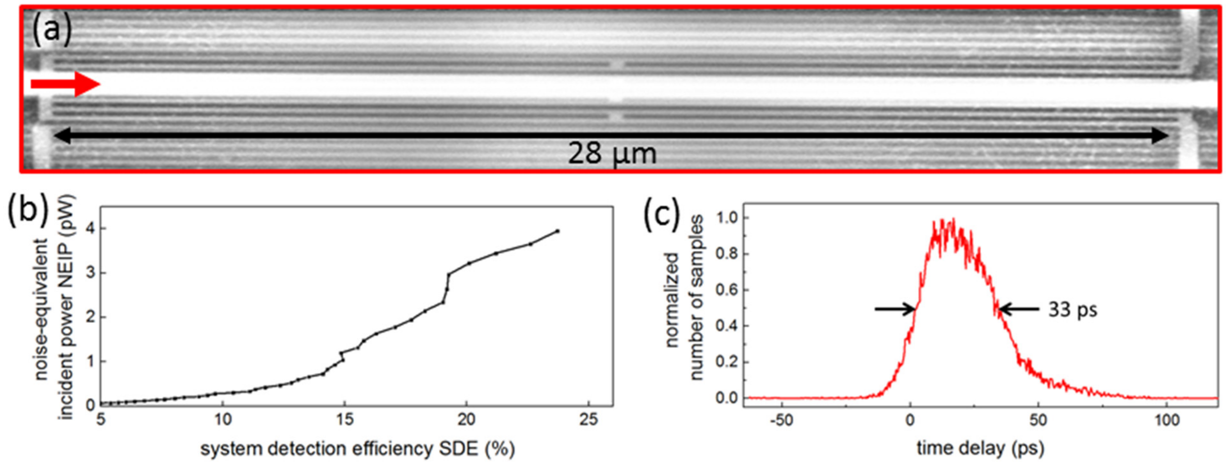


**Supplementary Figure 15.** (a) Top-down SEM of a membrane-detector integrated with a Si waveguide. The length of the detector is ~ 28 μm. (b) System detection efficiency (SDE) vs. noise-equivalent incident power for a directional coupler integrated with two large-coupling-length detectors as shown in (a). The relative error of the SDE values is ±10%. (c) Instrument response function of a waveguide-integrated detector measured with reduced length of the electrical path.

**Supplementary Table 1.** Calculated inductance values for series-2-SNAPs based on 80-nm-wide nanowires. These values were used to design the detectors. For each 2-SNAP, we need > 3*$L_{kin}$ of a single section in series to ensure that the detectors have a broad avalanche regime of at least 20% of the switching current of an unconstricted SNAP (see Supplementary Ref. [6] for more details). Since every 2-SNAP has already three 2-SNAPs in series (3*1/2* $L_{kin}$ of single section), we only need to add $L_S \geq 1.5$*$L_{kin}$ of a single section as series inductor.

| Nanowire width (nm) | Detector length (µm) | Nanowire length per SNAP section (µm) | #squares, $L_{kin}$ per section (nH) [assuming 80pH/square] | $L_S \geq$ 1.5 x $L_{kin}$ per section | Total inductance L of detector (nH) =$L_S$+2*($L_{kin}$ per section) | Estimated reset time =3*L/50Ω (ns) |
|---|---|---|---|---|---|---|
| 80 | 17 | 17 | 213, 17 | 50 [2.9x] | 84 | 5 |

## Supplementary Discussion

**Effect of membrane fabrication and thermal cycling on SNSPDs.** Before transferring membranes onto the PIC, we characterized the room-temperature resistance $R_{after}$ of detectors suspended on membranes and compared to detector resistance values $R_{before}$ before the substrate was removed. Supplementary Figure 13(a) shows that the relative detector resistance change ($R_{after}$ - $R_{before}$)/$R_{before}$ was 1-2%, indicating no significant material damage to the detectors due to the membrane fabrication process. Supplementary Figure 13(b) shows the critical current of membrane-detectors that were successfully transferred onto a secondary substrate. The membranes here consisted of ~300-nm-thick $SiN_x$. The critical currents of detectors on 300- to 400-nm-thick membranes were suppressed by ~10% compared to values measured on the solid substrate before undercut, while critical currents of detectors on sub-200-nm-thick membranes were suppressed by ~10-20%. Due to the small change in room temperature resistance values (Supplementary Figure 10(a)) we attribute the critical current suppression to the lower thermal capacity of the membranes compared to a solid substrate. Thermal cycling did not result in a measurable degradation (within the measurement accuracy of ~0.5 µA) of critical current of the transferred detectors.

**Dependence of system dark count rate on shielding conditions.** We used a closed-cycle cryostat with optical access to operate the chip shown in Figure 1(b) in the main text. The schematic cross-section of the cryostat is shown in Supplementary Figure 14(a). The PIC chip and platform holding the micro-manipulated lensed fibers were kept at 3 K base temperature. In order to couple light from the lensed fibers into the waveguides, the edges of the chip, containing the polymer couplers (Figures 1(c-I, c-II) in the main text), were imaged through the windows using a 50x long-working-distance objective. The direct imaging greatly simplified pre-alignment, while finer fiber-to-coupler alignment was performed using feedback from the on-chip detectors. However, the optical access ports in this prototyping setup resulted in radiation leakage and therefore increased the system dark count rate of the detectors significantly, as shown in Supplementary Figure 14(b). When we replaced the 30 K window in the cryostat with a solid copper plate, we observed a significantly lower dark count rate of ~ 5 kcps instead of ~ 800 kcps at the operation point.

**Improving detection efficiency and jitter.** For low-jitter detectors, the detection efficiency is limited by the optical absorption [7]. The optical absorption can be improved further by increasing the detector-to-waveguide coupling length. Supplementary Figure 15(a) shows a detector integrated with a 500-nm-wide silicon waveguide as outlined in the main text. This detector has a coupling length of 28 µm (compared with 16-17 µm for the detectors in the main text). We integrated these detectors with an on-chip directional coupler as shown in Supplementary Figure 1(c). Supplementary Figure 15(b) shows the system detection efficiency vs. noise-equivalent incident power for these waveguide-integrated detectors. The increased coupling length resulted in a system detection efficiency up to 24 ± 2%, an improvement by ~ 26 ± 3% compared to the previous detector design with shorter coupling length. The timing jitter of the waveguide-integrated detectors is limited by the signal-to-noise ratio of the detector pulse, which can be improved by decreasing losses in the RF lines. We demonstrated this limiting factor by reducing the length of the RF line by 1 cm and the length of our wire-bonds onto the Au pads by 3 mm. The shorter electrical path resulted in a FWHM timing jitter of 33 ps, as shown in Supplementary Figure 15(c), a significant improvement compared to ≥42 ps jitter previously measured in the same cryostat.

## Supplementary Methods

**Optical Simulations.** We used a finite-element model to calculate the length of the detector that would provide sufficient absorption of the mode travelling in the waveguide. The simulated geometry, superposed by the electromagnetic mode profile, is shown in Supplementary Figure 2(a). In this work, we consider only the fundamental TE mode; we experimentally observe no noticeable conversion from this mode to other waveguide modes. The detector was separated from the surface of the waveguide by residual resist layers used to fabricate the detector and the waveguide. We estimated the thickness of the residual resist layer as 20 to 80 nm. The 2D finite element simulations, performed in COMSOL, were used to calculate the imaginary part of the effective mode index $n_{\mathrm{i}}$. Following Supplementary Ref. [1], we calculated the optical absorption $\alpha$ in the detector as $\alpha = 1 - \exp(-4\pi \cdot n_{\mathrm{i}} \cdot L_{\mathrm{C}} / 1.55)$, where $L_{\mathrm{C}}$ is the detector (coupling) length in µm. Based on the calculated absorption in the detector, shown in Supplementary Figure 2(b), we chose $L_{\mathrm{C}} = 17$ µm to ensure $\alpha > 50$ %. In practice, we measured optical absorption values of 62 – 74 %. The optical absorption could be further increased by increasing $L_{\mathrm{C}}$ [2].

There are two main experimental factors besides detector length that limit the practically achievable detector absorption: misalignment and scattering at the interface of the $SiN_x$ detector membrane. We show in Supplementary Figure 3(a) the effect of misalignment on absorption. The detector region is 1.5 µm wide, but only nanowires directly above the waveguide have appreciable overlap with the waveguide mode. Therefore, within the detector active area, the absorption is roughly periodic as a function of horizontal displacement with a period of 200 nm, the nanowire pitch. Even at 100 nm misalignment the absorption is reduced by only 0.8%. For this simulation, we assumed 80 nm resist and a 200 nm $SiN_x$ membrane. We calculate the transmission $\kappa$ at the $SiN_x$ membrane edge using coupled mode theory. $\kappa$ is therefore calculated using an overlap integral defined as

$$\kappa = \left| \mathrm{Re}\left[ \frac{\left(\int \vec{\mathrm{E}}_1 \times \vec{\mathrm{H}}_2 \cdot d\vec{S}\right)\left(\int \vec{\mathrm{E}}_2 \times \vec{\mathrm{H}}_1 \cdot d\vec{S}\right)}{\int \vec{\mathrm{E}}_1 \times \vec{\mathrm{H}}_1 \cdot d\vec{S}} \right] \frac{1}{\mathrm{Re}\left[\int \vec{\mathrm{E}}_2 \times \vec{\mathrm{H}}_2 \cdot d\vec{S}\right]} \right|,$$

, which gives the fraction of power transmitted from the silicon waveguide with air cladding to the silicon waveguide with $SiN_x$ cladding. For our experimental conditions, $\kappa < 1\%$. The perturbation is this small

because the silicon nitride is thin, the index of silicon nitride is significantly lower than that of silicon, and the resist spacer layer on the silicon waveguide decreases the mode of overlap with the silicon nitride. We plot the mode pattern with and without the $SiN_x$ cladding in Supplementary Figure 3(b).

**Nanowire circuit.** The waveguide-detectors consisted of four units connected in series, with each unit comprising two ~80-nm-wide nanowires (200 nm pitch) in parallel. Detectors comprising this parallel-nanowire structure are commonly referred to as superconducting nanowire avalanche photodetectors (SNAPs [3, 4]). This detector design, illustrated in Supplementary Figures 1(a, b), is similar to detectors in Supplementary Ref. [5]. The value of the series inductor $L_S$ was generally chosen as ~ 50 nH so that the total inductance in series with a single parallel-nanowire unit was about 3- to 7-times the series inductance of a single nanowire (see Supplementary Ref. [6]). The detailed inductance values are listed in Supplementary Table 1.

**Membrane layout.** Supplementary Figure 4(a) shows a basic (initial) design of a suspended membrane-detector, connected to the bulk substrate via four ~15-μm-long microbridges. These bridges had an unpredictable breaking pattern (Supplementary Figure 4(b)), resulting in fractured $SiN_x$ pieces that could fall in between the membrane and the PIC chip surface and prevent tight contact between the detector and the waveguide chip. In order to avoid residual $SiN_x$ pieces we modified the bridge design as shown in Supplementary Figure 4(c): the bridges were shorter (~3 μm long) with a ~0.8- to 1.5-μm-long constriction in the middle section of the bridge, resulting in a preferred breaking region marked by the dashed red lines. With this improved design most membranes could be removed from the bulk substrate (Supplementary Figure 4(d)) without substantial residual $SiN_x$ pieces.

**Resist preparation and cleaning.** During the membrane-detector fabrication process, illustrated in Supplementary Figure 5, photoresist layers covering the detector are used to define the outline of the membrane with trenches (Supplementary Figure 5(b)) and to protect the detector during the Si etch step (Supplementary Figure 5(c)). Initially the protective etch mask that was used to fabricate the trenches via reactive ion etch (RIE) with $CF_4$ was also used as a protective layer in the subsequent etch step with $XeF_2$. The fluorine gas (plasma) treatment during the RIE fluorinated the surface and hard-baked the resist, making it irremovable in solvents unless ultrasonic agitation was used. However, sonication could not be used after membrane undercut since it was found to cause membrane collapse. Oxygen-helium plasma (ashing) was the remaining option, but we could not remove the hard-baked residue after ashing, shown in Supplementary Figure 9(b). We solved this issue by removing the resist mask after the trenches were fabricated via sonication (‘photoresist 1’ in Supplementary Figure 5(b)), and coating the detectors with a new resist mask for the silicon removal step (‘photoresist 2’ in Supplementary Figure 5(c)). Since the second mask was not exposed to a long $CF_4$ etch, we were able to remove it in an NMP-based resist stripper followed by an acetone and IPA rinse. While requiring an additional photolithography step, this stripping process did not leave a visible residue on the nanowires, as shown in Supplementary Figure 12(c).

## Supplementary References

[1] X. Hu, C. Holzwarth, D. Masciarelli, E. Dauler, and K. Berggren, “Efficiently Coupling Light to Superconducting Nanowire Single-Photon Detectors,” IEEE Transactions on Applied Superconductivity **19**, pp. 336-340 (2009).

[2] V. Kovalyuk, W. Hartmann, O. Kahl, N. Kaurova, A. Korneev, G. Goltsman, and W. Pernice, “Absorption engineering of NbN nanowires deposited on silicon nitride nanophotonic circuits,” Optics Express **21**(19), pp. 22683-22692 (2013).

[3] M. Ejrnaes, R. Cristiano, O. Quaranta, S. Pagano, A. Gaggero, F. Mattioli, R. Leoni, B. Voronov and G. Gol’tsman, “A cascade switching superconducting single photon detector,” Applied Physics Letters **91**, 262509 (2007).

[4] F. Marsili, F. Najafi, E. Dauler, F. Bellei, X. Hu, M. Csete, R. Molnar, and K. Berggren, “Single-Photon Detectors Based on Ultranarrow Superconducting Nanowires,” Nano Letters **11**(5), 2048 (2011).

[5] M. Ejrnaes, A. Casaburi, O. Quaranta, S. Marchetti, A. Gaggero, F. Mattioli, R. Leoni, S. Pagano and R. Cristiano, “Characterization of parallel superconducting nanowire single photon detectors,” Superconducting Science and Technology **22**, 055006 (2009).

[6] F. Marsili, F. Najafi, E. Dauler, R. Molnar, K. Berggren, “Afterpulsing and Instability in Superconducting Nanowire Avalanche Photodetectors,” Applied Physics Letters **100**, 112601 (2012).

[7] W. Pernice, C. Schuck, O. Minaeva, M. Li, G. Gol’tsman, A. Sergienko and H. Tang, “High-speed and high-efficiency travelling wave single-photon detectors embedded in nanophotonic circuits,” Nature Communications **3**, 1325 (2012).
